# Enhanced Seroconversion to West Nile Virus Proteins in Mice by West Nile Kunjin Replicon Virus-like Particles Expressing Glycoproteins from Crimean–Congo Hemorrhagic Fever Virus

**DOI:** 10.3390/pathogens11020233

**Published:** 2022-02-10

**Authors:** Pham-Tue-Hung Tran, Urban Höglund, Olivia Larsson, Sofia Appelberg, Ali Mirazimi, Magnus Johansson, Wessam Melik

**Affiliations:** 1 School of Medical Science, Inflammatory Response and Infection Susceptibility Centre (iRiSC), Örebro University, 703 62 Örebro, Sweden; hung.tran@oru.se (P.-T.-H.T.); magnus.johansson@oru.se (M.J.); 2 Adlego Biomedical AB, P.O. Box 42, 751 03 Uppsala, Sweden; urban.hoglund@adlego.se (U.H.); olivia.larsson@adlego.se (O.L.); 3 Department of Microbiology, Public Health Agency of Sweden, 171 82 Solna, Sweden; sofia.appelberg@folkhalsomyndigheten.se; 4 Division of Clinical Microbiology, Department of Laboratory Medicine, Karolinska Institution, 141 52 Huddinge, Sweden; ali.mirazimi@ki.se; 5 National Veterinary Institute, 751 89 Uppsala, Sweden

**Keywords:** replicon virus-like particles (RVPs), replicons, West Nile Kunjin virus, envelope, non-structural protein 1, Crimean–Congo hemorrhagic fever virus, glycoprotein (Gn–Gc), seroconversion, neutralization, vaccines

## Abstract

Removal of genes coding for major parts of capsid (C), premembrane (prM), and envelope (E) proteins on the flavivirus genome aborts the production of infectious virus particles where the remaining genome forms a replicon that retains replicability in host cells. The C-prM-E proteins can also be expressed in *trans* with the flavivirus replicons to generate single-round infectious replicon virus-like particles (RVPs). In this study, we characterized the use of RVPs based on the Kunjin strain of WNV (WNV_KUN_) as a putative WNV vaccine candidate. In addition, the WNV_KUN_ C-prM-E genes were substituted with the Crimean–Congo hemorrhagic fever virus (CCHFV) genes encoding the glycoproteins Gn and Gc to generate a WNV_KUN_ replicon expressing the CCHFV proteins. To generate RVPs, the WNV_KUN_ replicon was transfected into a cell line expressing the WNV_KUN_ C-prM-E. Using immunoblotting and immunofluorescence assays, we showed that the replicon can express the CCHFV Gn and Gc proteins and the RVPs can transduce cells to express WNV_KUN_ proteins and the CCHFV Gn and Gc proteins. Our study also revealed that these RVPs have potential as a vaccine platform with low risk of recombination as it infects cells only in one cycle. The immunization of mice with the RVPs resulted in high seroconversion to both WNV E and NS1 but limited seroconversion to CCHFV Gn and Gc proteins. Interestingly, we found that there was enhanced production of WNV E, NS1 antibodies, and neutralizing antibodies by the inclusion of CCHFV Gc and Gn into WNV_KUN_ RVPs. Thus, this study indicates a complementary effect of the CCHFV Gn and Gc proteins on the immunogenicity by WNV_KUN_ RVPs, which may be applied to develop a future vaccine against the WNV.

## 1. Introduction

The neurotropic West Nile virus (WNV) consists of many lineages, among which the lineage 1 has been associated with the majority of outbreaks [1]. In this group, infections of the New York 1999 strain (WNV_NY99_) can result in severe meningoencephalitis with a fatality rate around 1% [2]. However, there is no approved human vaccine or antiviral treatment for the infection. In the same lineage, infections of the Kunjin strain (WNV_KUN_), a naturally attenuated WNV, has resulted in only a few nonfatal disease cases since 1960 [3,4]. Furthermore, WNV_NY99_ and WNV_KUN_ share 89% of the envelop (E) gene, 88% of the non-structure (NS) 5 gene and the 3’ untranslated region (UTR) [3]. Therefore, WNV_KUN_ could be used as a strategy to develop a vaccine against WNV_NY99._

WNV_NY99_ and WNV_KUN_ are members of the family *Flaviviridae*, which are enveloped viruses with icosahedral structures. The viral genome, which is an approximately 11 kb positive-sense single-stranded RNA (+ssRNA), codes for a single polyprotein. Cleavages of the polyproteins by viral and cellular proteases result in three structural proteins—capsid (C), precursor membrane (prM), and E for viral assembly, as well as seven NS proteins. In addition, the viral genomic RNA is flanked by 5′- and 3′-UTRs which are required for translation, replication, and assembly [5,6,7,8].

The removal of the C-prM-E genes in the flavivirus genome results in replicons that can replicate in transfected cells but abolish production of infectious virus particles [9,10,11,12]. WNV_KUN_ was one of the first flavivirus replicon systems constructed [13]. The replicon can be co-expressed with the corresponding structural genes, resulting in the packaging of replicon and the generation of replicon virus-like particles (RVPs). Several studies have demonstrated the potential of flavivirus RVPs as a vaccine platform [14,15,16,17]. 

Crimean–Congo hemorrhagic fever virus (CCHFV) is a highly virulent virus that has a fatality rate between 5 and 30%, depending on the outbreaks [18]. As with other members of the genus *Orthonairovirus* and family *Nairoviridae*, the virus is an enveloped negative-sense RNA virus. The virus has three genomic segments: small, medium, and large encoding nucleoproteins; glycoproteins; and RNA-dependent RNA polymerase, respectively. The virus infects humans mainly through *Hyalomma* ticks, which live throughout Africa, Southern and Eastern Europe, the Middle East, India, and Asia [19]. 

As flavivirus RVPs can be used as a vector to transduce cells and express proteins of interest, they are potentially employed as a multiple vaccine platform. Though CCHFV and WNV infect human and livestock by different vectors, i.e., tick and mosquito, respectively, there are overlapping geographic distributions of the two viruses in Western Asia and Balkan Europe [20,21,22,23]. In this study, we generated WNV_KUN_ RVPs that deliver genes coding glycoproteins CCHFV Gn and Gc to infected cells. These RVPs were then administered to mice to examine their potential as a vaccine candidate. The administration of RVPs into mice induced seroconversion, generating antibodies against CCHFV Gn, CCHFV Gc, WNV_NY99_ NS1, and WNV_NY99_ E. However, serum from the CCHFV Gn-Gc RVP-injected mice limitedly neutralize CCHFV but enhanced the neutralization of WNV_KUN_ and seroconversion to WNV_NY99_ NS1 and E. The data in this study highlight a strategy using the WNV_KUN_ RVPs with the CCHFV Gn-Gc as a vaccine against WNV.

## 2. Results

### 2.1. CCHFV Gn–Gc Expression by the WNV_KUN_ Replicon

**Figure 1:**
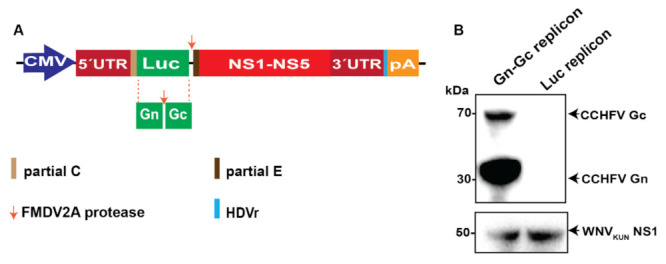
Expression of Crimean–Congo hemorrhagic fever virus (CCHFV) glycoproteins Gn and GC proteins using the West Nile Kunjin (WNV_KUN_) replicon. (**A**) The reporter luciferase (Luc) gene was substituted with genes encoding CCHFV Gn and Gc in the WNV_KUN_ DNA replicon. In short, the replicon is driven by the cytomegalovirus (CMV) promoter expressing an open reading frame flanked by the 5′- untranslated region (UTR) and the 3′-UTR comprising: first, partial capsid (C) gene fused in frame with the Luc, the foot and mouth disease virus autoprotease 2a (FMDV2A) then partial envelop (E) gene, and all the nonstructural proteins. The hepatitis delta virus antigenomic ribozyme (HDVr) sequence was inserted immediately downstream of the WNV_KUN_ 3′-UTR followed by the Simian virus 40 (SV40) polyadenylation signal (pA). (**B**) Immunoblotting of cell lysates 2 days after transfection of the WNV_KUN_ CCHFV Gn–Gc replicons versus the WNV_KUN_ Luc replicon into the BHK-21 cell line expressing the WNV_KUN_ C–prM–E.

As we aimed to generate mutivalent WNV_KUN_ RVPs that can transduce the CCHFV Gn–Gc gene, we initially generated a DNA WNV_KUN_ replicon that can express the CCHFV glycoproteins Gn and Gc, termed Gn–Gc replicon. Here, the luciferase (Luc) reporter gene from the previously described WNV_KUN_ replicon [24] was substituted with genes encoding CCHFV Gn–Gc with the foot-and-mouth disease virus autoprotease 2a (FMDV2A) gene inserted between Gn and Gc, allowing for the cleavage of the Gn–Gc into Gn and Gc during replicon expression (Figure 1A). The replicons were then transfected into BHK-21 cells stably expressing WNV_KUN_ C-prM-E, as described previously [24]. Compared to the Luc replicon, the Gn–Gc replicon expressed the CCHFV Gn and Gc protein as expected (Figure 1B). In addition, both replicons also expressed the WNV_KUN_ NS1 protein (Figure 1B). All together, these suggest the replicons can express the WNV_KUN_ polyprotein and the inserted CCHFV Gn and Gc genes.

### 2.2. WNV_KUN_ RVPs Transduced Their Polyprotein Gene and CCHFV Gn–Gc Gene in A Single Cycle

**Figure 2:**
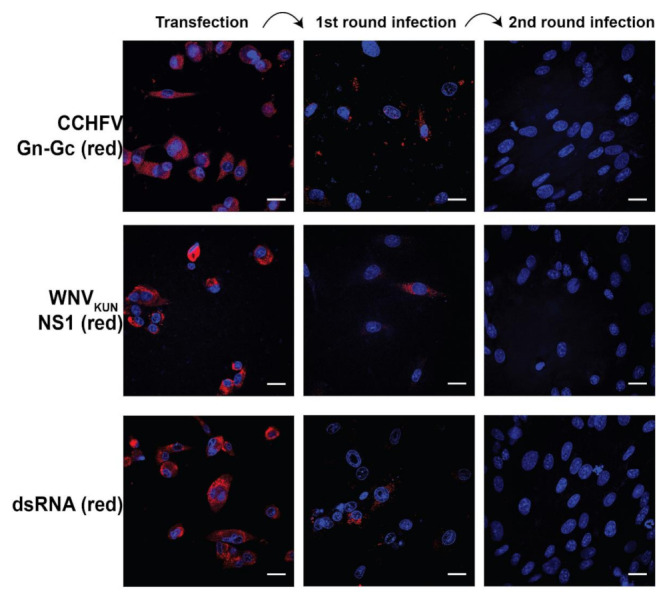
Immunofluorescence labeling of BHK-21 C-prM-E cells after transfection with the Gn–Gc replicon, followed by two consecutive cycles of RVP infection into naïve BHK-21 cells. The cells were visualized with the antibodies anti-CCHFV Gn–Gc (red), anti-WNV_KUN_ NS1 (red), and anti-dsRNA (red). The nucleus was counterstained with DAPI (blue). Bar scales represent 20 µm.

As the Gn–Gc replicon could express the Gn and the Gc proteins, we characterized the packaging of the replicon by the C-prM-E proteins to generate RVPs and its safety. We initially transfected the Gn–Gc replicon into the WNV_KUN_ C-prM-E BHK-21 cell line as previously described [24]. Here, CCHFV Gn, Gc, and WNV_KUN_ NS1 could be visualized, indicating replicon expression, and staining of dsRNA revealed replicon replication (Figure 2).

As the cell line can express the C-prM-E proteins, the replicon can be encapsidated and enveloped to generate RVPs. To characterize this postulation, we collected the supernatant from transfected cells and infected naïve BHK-21 cells in two consecutive cycles. Media from the RVP-generating cells were diluted to the dilutions with the highest numbers of cells infected. In the first round of infection, cells were transduced to express the CCHFV Gn, Gc, and WNV_KUN_ NS1 proteins, and immunostainings revealed replicon replication (Figure 2). Because naïve BHK-21 cells do not have the gene encoding C-prM-E, the produced replicon should not be able to be packaged to form new RVPs. As expected, BHK-21 infected in the second cycle could not express the replicon. These results indicate that the Gn–Gc replicon can be packaged to generate RVPs and that the RVPs infect cells only in a single cycle, similarly to the Luc RVPs [24].

### 2.3. CCHFV Gn–Gc RVPs Induced Seroconversion to CCHFV Gn and Gc Proteins and Enhanced Seroconversion to WNV_NY99_ NS1 and E Proteins

**Figure 3:**
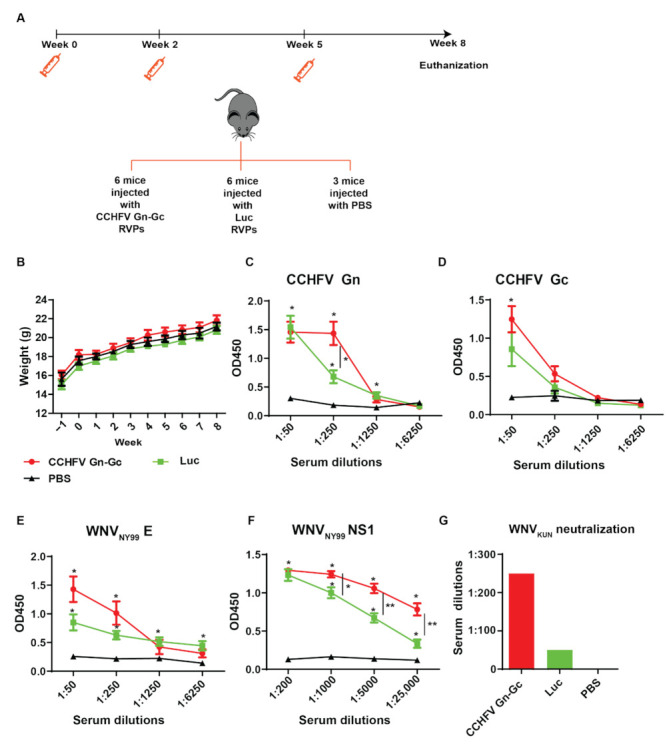
CCHFV Gn–Gc RVPs induced seroconversion to CCHFV Gn and Gc and enhanced seroconversion to WNV_NY99_ NS1, E. (**A**) Schematic illustration of the mice immunization schedule. Mice were subcutaneously immunized three times at weeks 0, 2, and 5 with RVPs expressing CCHFV Gn–Gc (6 mice), Luc (6 mice), or phosphate-buffered saline (PBS) (3 mice). (**B**) Mouse weight from one week before the experiment to the mouse-euthanized day. Mice sera from the study groups were diluted and assayed with enzyme-linked immunosorbent assays (ELISA) to measure antibody titers against CCHFV Gn (**C**), CCHFV Gc (**D**) WNV_NY99_ E (**E**), and WNV _NY99_ NS1 (**F**). The end-point titers were determined as there was no difference in the measured optical density values at 450 nm (OD450) between the vaccinated group and the control group. The experiments were conducted with two technical repeats. The p values are indicated using * *p* < 0.05 and ** *p* < 0.01. (**G**) Serum titers that elicited 50% reduction in the WNV_KUN_ plaque number. Sera from experimented animal were combined before assaying.

As we demonstrated that RVPs could transduce cells in vitro, we examined the in vivo immunogenicity by administering them into mice. Mice were divided into three groups and subcutaneously injected with either Gn–Gc RVPs, Luc RVPs, or PBS (Figure 3A). During the study, mice from all groups were healthy and showed no significant differences in weight (Figure 3B).

We then determined the antibody titers in sera. Mice immunized with CCHFV Gn–Gc RVPs showed seroconversions to Gn and Gc proteins with the serum titers reaching 1:250 and 1:50 dilutions, respectively (Figure 3C,D). As there were seroconversions to Gn and Gc proteins by mice injected with the control Luc RVPs, there might be cross-reactivity of WNV_KUN_ RVPs antibodies to the Gn–Gc protein. 

As the WNV_KUN_ replicon is packaged by C-prM-E to generate RVPs and the WNV_KUN_ is the attenuated strain of WNV_NY99_ [3,4], we determined the seroconversion of the RVPs to the WNV_NY99_ E and NS1. Mice immunized with either Gn–Gc RVPs or Luc RVPs showed seroconversion to the WNV_NY99_ E and NS1, whose antibody titers can reach to more than 1:6250 and 1:25,000 dilutions, respectively (Figure 3E,F). Interestingly, there was a significant enhancement of seroconversion to these proteins in mice immunized by the Gn–Gc RVPs, compared to the Luc RVPs.

Ultimately, we examined virus neutralization by sera. As WNV_KUN_ share 89% of the envelop (E) gene to WNV_NY99_ [3] and the WNV_KUN_ virus in a neutralization assay is often employed in the context of WNV_NY99_ vaccine development [25,26], neutralization to WNV_KUN_ by the sera was examined. As expected, pooled sera from all mice administrated with the Gn–Gc RVPs showed the enhanced effect of neutralizing the WNV_KUN_, as the antibody titer with a 50% reduction in plaque numbers was 1:250, compared to 1:50 in mice administered with Luc RVPs (Figure 3G). 

To eliminate the effect of cross-reactivity, we examined CCHFV neutralization by the sera from the Gn–Gc RVP group compared to the Luc RVP group. Sera from the Gn–Gc RVPs were divided into two groups based on a high or low level of antibodies before assaying. In accordance with the antibody levels of the two groups, at the first serum dilution (1:8), diluted sera from the mice with a higher Gc antibody level and these from the mice with a lower Gc antibody level showed around 35% and 15% neutralization of infection, respectively, compared to sera from the control (RVPs-Luc) immunized mice (data not shown). 

These data suggest that the RVPs can induce seroconversion to antibodies against the CCHFV and WNV_NY99_ proteins. In addition, the CCHFV Gn–Gc can enhance the seroconversion to the WNV_NY99_ proteins, suggesting that they can be incorporated to the WNV_KUN_ RVPs, enhancing the potentials of the RVPs as a putative vaccine candidate against WNV.

## 3. Discussion

Since the last outbreak in New York 1999, WNV_NY99_ has caused nearly 25,000 cases with neuronal invasive symptoms (encephalitis and meningitis) and more than 2000 deaths in North America [2]. In Europe, the virus has caused more than 2000 disease cases and 181 deaths [27]. Thus, there is need to have approved vaccines against the virus, preventing future pandemic burden. There have been six vaccine candidates against WNV in the clinical trial phases I and II, including DNA vector expressing WNV_NY99_ E, recombinant WNV_NY99_ E protein, inactivated WNV_NY99_, inactivated WNV_KUN_, live chimeric WNV_NY99_–dengue 4 virus, and live chimeric WNV_NY99_–yellow fever virus 17D [28,29]. 

As WNV_NY99_ neurological disease is more severe in the elderly, the major target of vaccines against the virus should be the immunosenescent population. In this context, the immunogenicity and safety of the vaccine are essential. The WNV_KUN_ RVP platform is based on the WNV_KUN_ virus, which is an attenuated strain of WNV [3,4]_._ As the particle contains replicons with no viral packaging genes, it can infect cells in a single cycle, abolishing systemic infection. As a strategy for vaccine development, it is a compromise between inactivated and live-attenuated virus vaccines. Unlike inactivated virus particles, WNV_KUN_ RVPs can preferentially infect and transduce antigen-presenting cells, such as macrophages or dendritic cells [30,31,32,33], and express vaccine candidate proteins, therefore inducing a greater immune response. Compared to live-attenuated virus vaccines, the infectivity of RVPs is limited to a single round, which makes them a safer vaccine platform for the immunosenescent population.

In this study, we characterized the expression and the safety of WNV_KUN_ RVPs, which deliver CCHFV Gn and Gc. Without the CCHFV nucleoprotein, these two proteins cannot function as an CCHFV packaging system. Thus, there is no risk for generation of a chimeric WNV_KUN_–CCHFV virus using the RVP platform. Indeed, we showed that the RVPs with these genes only infected cells in a single round and mice injected with the RVP survived. Immunization of the RVPs elicited seroconversions to the antibodies in mice. The Gn–Gc RVPs also conferred the production of antibodies against the WNV_NY99_ NS1 and E, given that WNV_KUN_ is an attenuated strain of WNV_NY99_. Interestingly, the expression of CCHFV Gn and Gc enhanced the immunogenicity of the WNV_NY99_ E, NS1, and WNV_KUN_ neutralization, which requires further investigation. Indeed, many epitopes within the CCHFV Gn and Gc have been predicted to induce an immune response to CD8^+^, CD4^+^, and linear B cells, which may suggest the enhanced antibody production to WNV_NY99_ E and NS1 by Gn and Gc [34,35].

Furthermore, the Gn–Gc RVPs from this study could not induce strong neutralizing antibodies against CCHFV. Likewise, the neutralizing antibody response from CCHFV survivors is also usually low, ranging from 1:8 to 1:32 according to fluorescent focus reduction tests [36]. Interestingly, epitope mapping from CCHFV survivors indicated that antibody responses toward epitopes within Gn and Gc are not likely to elicit neutralizing antibodies [37], but passive immunization with these antibodies was able to protect against CCHFV, highlighting the roles of other mechanisms, such as antibody-dependent cell-mediated cytotoxicity. Thus, although mice sera did not strongly induce CCHFV neutralization, future studies should examine the T cell response to the CCHFV by the Gn–Gc RVPs.

## 4. Materials and Methods

### 4.1. Cell Culture

Baby hamster kidney (BHK-21) (ATCC) and Vero (ATCC) cells were maintained in Dulbecco’s modified Eagle’s medium (DMEM) containing 1 g/L of glucose (Gibco, Paisley, UK), supplemented with 10% heat-inactivated fetal bovine serum (HI–FBS) (Gibco) and 100 U/mL of penicillin–streptomycin (PEST) (Gibco) at 37 °C in 5% CO_2_.

### 4.2. Preparation of Gene Constructs

The WNV_KUN_ replicon was constructed based on the WNV_KUN_ sequence (accession number AY274504), as described previously [24]. To generate the WNV_KUN_ replicon expressing the CCHFV Gn and Gc, the CCHFV Hoti strain Gn and Gc genes were PCR-amplified from the M segment cDNA clone (accession number MH483985.1) with suitable primers to create a FMDV2A between the Gn and Gc and suitable restriction sites flanking the Gn–Gc. The fragment was then substituted with the Luc gene in the WNV_KUN_ replicon by suitable restriction reactions and ligations.

### 4.3. Proteins and Antibodies

The following proteins and antibodies were used in this study: the WNV_NY99_ E protein, the WNV_NY99_ NS1 protein, the CCHFV Gn protein, the CCHFV Gc protein human Fc tag, the mouse anti-CCHFV Gn (The Native Antigen Company, Oxford, UK), the mouse anti-flavivirus NS1 (Abcam, Cambridge, UK), the mouse J2 anti dsRNA (Scicons, Szirák, Hungary), the mouse anti-CCHFV Gn–Gc (Friedrich-Loeffler-Institute, Greifswald, Germany), the Alexa Fluor 594-conjugated anti-mouse goat antibody (Invitrogen, Vilnius, Lithuania), and the HPR-conjugated anti-mouse goat antibody (Invitrogen).

### 4.4. Protein Electrophoresis and Immunoblotting

Protein electrophoresis and immunoblotting were conducted as previously described [24,38]. RIPA buffer (Thermoscientific, Waltham, MA, USA) with protease inhibitors (Sigma, St. Louis, MO, USA) was used to lyze cells for 20 min at 4 °C, followed by boiling in LDS sample buffer (Invitrogen). Proteins were separated for 70 min at 120 V constant on precast 4–12% polyacrylamide Bis–Tris gels in MES running buffer (Invitrogen), followed by transfer to nitrocellulose membranes using the iBlot 2 Gel Transfer Device (Invitrogen). Proteins of interest were detected with the antibodies anti-CCHF Gn (1:1000), anti-CCHFV Gn–Gc (1:100), and anti-flavivirus NS1 (1:100).

### 4.5. Immunofluorescence Assay

Cells were fixed by 4% paraformaldehyde (Scharlau, Barcelona, Spain) for 20 min at room temperature (RT). They were then permeabilized by 0.1% Triton X-100 (VWR), followed by blocking with 2% bovine serum albumin (Fitzgerald, MA, USA) and 2% goat serum (Invitrogen). Cells were labeled with the primary antibody anti-CCHFV Gn–Gc (1:100) for 2 h at RT, followed by an incubation with the secondary antibody Alexa Fluor 594 (1:500) for 1 h at RT. Images were captured using a confocal laser scanning microscopy SP8 (Leica, Wetzlar, Germany) and analyzed using ImageJ.

### 4.6. RVP Purification and RVP Concentration Measurement

The RVPs were purified as previously described [24]. In short, supernatants from the RVP production system were collected then loaded on 25% sucrose (Sigma), following by ultra-centrifugations. After centrifugation, both the supernatant and the sucrose were removed, and the RVPs were dissolved in DMEM. To monitor RVP concentration, diluted RVPs were utilized to infect confluent BHK-21 cells for 1 h at 37 °C in 5% CO_2_ incubator. The cells were immunofluorescent labeled two days after the infection to count the number of infected cells revealing the number of RVPs. 

### 4.7. Animal Immunizations

Six-week-old female BALB/cN mice (Charles River, Freiburg, Germany) were administrated subcutaneously with 200 µL of concentrated RVPs (approximately 10^6^ particles). Mice were divided into three groups and administrated: RVPs carrying replicons expressing CCHFV Gn–Gc (six mice), RVPs carrying replicons expressing Luc (six mice), or phosphate-buffered saline (PBS) (Gibco).

### 4.8. Sample Collection

Three weeks after the last RVP immunization, the mice were anesthetized with isoflurane for collection of retro-orbital blood samples, after which they were euthanized. The blood was incubated at RT for 30 min before centrifugation at 2000× *g* for 10 min at 20 °C. The sera were collected and stored at −80 °C until use.

### 4.9. Enzyme-Linked Immunosorbent Assays (ELISA)

To detect antibodies against CCHFV Gn, Gc, WNV_NY99_ NS1, and E in sera, an in-house ELISA was developed using Nunc Maxisorb plates (Invitrogen) coated with the CCHFV Gn, Gc, WNV_NY99_ NS1, or E proteins at RT overnight. The plates were then blocked with PBS with 1% BSA for 2 h at RT and washed three times with PBST. Next, 100 µL of diluted sera were added to each well and incubated for 1 h at RT on a shaker at 700 rpm. After washing, horseradish peroxidase (HRP)-conjugated anti-mouse antibody (1:10,000) was added and incubated at RT for 1 h. After washing, 100 µL of substrate solution (ABcam), containing tetramethylbenzidine and peroxide, was added. Plates were then incubated for 15 min before adding 100 µL of stopping solution containing 1M of HCl. The absorbance was read at 450 nm using the Cytation 3 Multi-Mode Reader (BioTek, Bad Friedrichshall, Germany).

### 4.10. WNV_KUN_ and CCHFV Neutralization Assays

Sera collected from each group were pooled and heat-inactivated by incubation at 56 °C for 30 min before being 5-fold serially diluted in DMEM. WNV_KUN_ was rescued from infectious clones, as previously described [38], and was diluted so that there were approximately 50 infectious particles per volume. Next, the diluted sera were incubated for 1 h at 37 °C before infecting 90% confluent Vero cells growing on 24 well-plates for 1 h at 37 °C in 5% CO_2_. After the infection, cells were overlaid with DMEM supplemented with 1.2% Avicel (FMC, Philadelphia, PA, USA), 2% HI–FBS, 1X nonessential amino acids (Gibco), and 1% PEST. After 3 days, the overlays were removed, and cells were fixed by methanol (Fisher, Trinidad and Tobago) for 20 min. The fixed cells were stained with staining buffer containing 2% crystal violet (Sigma), 20% methanol, and 0.1% ammonium oxalate (Sigma) solution for 1 h before washing in water.

The titer of CCHFV neutralizing antibodies in the serum of immunized mice was determined by microneutralization assay. The CCHFV strain Kosova Hoti (07v-EVA70) was obtained from the European Virus Archive. Sera from all mice in the Luc RVPs or the PBS control group were pooled, and sera from mice immunized with CCHFV Gn–Gc RVPs were divided into two groups (high or low anti-CCHFV Gn–Gc antibodies). Thereafter, the sera were heat-inactivated for 30 min at 56 °C. Serial 2-fold dilutions of sera were mixed with 200 CCHFV Hoti viral particles and incubated at 37 °C for 1 h. Thereafter, 100 μL of the serum–virus mix was added in duplicate to Vero cells on a 96-well plate (20,000 cells/well). After 1 h incubation at 37 °C, the inoculum was removed, and cells were washed 3 times with DMEM supplemented with 2% FBS. Then, 100 μL of DMEM supplemented with 2% FBS was added, and the cells were incubated for 24 h at 37 °C and 5% CO_2_. Cells were fixed with an ice-cold acetone–methanol mix (1:1) in −20 °C overnight and stained for CCHFV nucleoprotein for enumeration of fluorescent foci. Total numbers of infected cells in each well were counted and the result was expressed as percent neutralization compared to infection in wells with serum from the control group for each dilution.

### 4.11. Statistics

Statistical differences between the means of parametric data (weights) were determined using two-way ANOVA, followed by Bonferroni correction, while statistical differences between non-parametric data (absorbance values) were determined using Mann–Whitney test. GraphPad Prism 9 was used to perform all statistical analyses. The values are presented as mean ± standard error of the mean. 

## 5. Conclusions

In conclusion, this study has shed new light on ways to improve the WNV_KUN_ RVPs as a vaccine platform against WNV. Our results suggest that the WNV_KUN_ RVPs have the capacity to transduce cells to express CCHFV Gn and Gc. The RVPs also enhanced the seroconversion and the production of neutralizing antibodies against WNV, which can increase the potential of the RVPs as a vaccine strategy against WNV.

## Data Availability

The data presented in this study are available in this article. Remaining data supporting reported results are available from the corresponding authors upon reasonable requests.
